# Addendum: Molecular Generation for Desired Transcriptome Changes With Adversarial Autoencoders

**DOI:** 10.3389/fphar.2020.01236

**Published:** 2020-08-21

**Authors:** Rim Shayakhmetov, Maksim Kuznetsov, Alexander Zhebrak, Artur Kadurin, Sergey Nikolenko, Alexander Aliper, Daniil Polykovskiy

**Affiliations:** ^1^Insilico Medicine, Hong Kong, Hong Kong; ^2^Neuromation OU, Tallinn, Estonia

**Keywords:** deep learning, generative models, adversarial autoencoders, conditional generation, representation learning, drug discovery, gene expression

In the original article, we missed the parallel work by Méndez-Lucio et al. (2020). This work also tackles a similar problem of generating molecular structures from transcriptomic data. The authors proposed a conditional model based on the generative adversarial networks Goodfellow et al. (2014). Unlike their approach, our model is joint, allowing us to generate molecular structures for a given gene expression profile and vice versa.
